# Standardization for Ki-67 Assessment in Moderately Differentiated Breast Cancer. A Retrospective Analysis of the SAKK 28/12 Study

**DOI:** 10.1371/journal.pone.0123435

**Published:** 2015-04-17

**Authors:** Zsuzsanna Varga, Estelle Cassoly, Qiyu Li, Christian Oehlschlegel, Coya Tapia, Hans Anton Lehr, Dirk Klingbiel, Beat Thürlimann, Thomas Ruhstaller

**Affiliations:** 1 Institute of Surgical Pathology, University Hospital Zurich, Zurich, Switzerland; 2 Swiss Group for Clinical Cancer Research, Bern, Switzerland; 3 Institute of Pathology, Cantonal Hospital St. Gallen, St. Gallen, Switzerland; 4 Institute of Pathology, University Bern, Bern, Switzerland; 5 Pathology Institute Friedrichshafen, Friedrichshafen, Germany; 6 Breast Center, Cantonal Hospital St. Gallen, St. Gallen, Switzerland; ACTREC, Tata Memorial Centre, INDIA

## Abstract

**Background:**

Proliferative activity (Ki-67 Labelling Index) in breast cancer increasingly serves as an additional tool in the decision for or against adjuvant chemotherapy in midrange hormone receptor positive breast cancer. Ki-67 Index has been previously shown to suffer from high inter-observer variability especially in midrange (G2) breast carcinomas. In this study we conducted a systematic approach using different Ki-67 assessments on large tissue sections in order to identify the method with the highest reliability and the lowest variability.

**Materials and Methods:**

Five breast pathologists retrospectively analyzed proliferative activity of 50 G2 invasive breast carcinomas using large tissue sections by assessing Ki-67 immunohistochemistry. Ki-67-assessments were done on light microscopy and on digital images following these methods: 1) assessing five regions, 2) assessing only darkly stained nuclei and 3) considering only condensed proliferative areas (‘hotspots’). An individual review (the first described assessment from 2008) was also performed. The assessments on light microscopy were done by estimating. All measurements were performed three times. Inter-observer and intra-observer reliabilities were calculated using the approach proposed by Eliasziw et al. Clinical cutoffs (14% and 20%) were tested using Fleiss’ Kappa.

**Results:**

There was a good intra-observer reliability in 5 of 7 methods (ICC: 0.76–0.89). The two highest inter-observer reliability was fair to moderate (ICC: 0.71 and 0.74) in 2 methods (region-analysis and individual-review) on light microscopy. Fleiss’-kappa-values (14% cut-off) were the highest (moderate) using the original recommendation on light-microscope (Kappa 0.58). Fleiss’ kappa values (20% cut-off) were the highest (Kappa 0.48 each) in analyzing hotspots on light-microscopy and digital-analysis. No methodologies using digital-analysis were superior to the methods on light microscope.

**Conclusion:**

Our results show that all methods on light-microscopy for Ki-67 assessment in large tissue sections resulted in a good intra-observer reliability. Region analysis and individual review (the original recommendation) on light-microscopy yielded the highest inter-observer reliability. These results show slight improvement to previously published data on poor-reproducibility and thus might be a practical-pragmatic way for routine assessment of Ki-67 Index in G2 breast carcinomas.

## Introduction

Proliferative activity and the use of genomic tests and their scores are getting increasing attention as they can be considered as further diagnostic tool additionally to traditional clinic-pathological parameters obtained on routine histological examination of surgically resected breast cancer specimens [1–3]. Especially midrange hormone receptor positive breast cancers may pose diagnostic challenges, as the indication for or against adjuvant chemotherapy cannot be met upon traditional clinic-pathological parameters in all instances [1–3]. The use of proliferative activity in hormone receptor positive breast cancers, measured by immunohistochemical assessment of the Ki-67 antigen was previously suggested on a study conducted on a BIG-1-98 patient cohort [4, 5]. This study suggested that Ki-67 labeling index (LI) above 14% is a potential marker in the decision for adjuvant chemotherapy, as Ki-67 LI >14% may identify patients who may benefit from adjuvant chemotherapy [5]. In the meantime, the assessment of Ki-67 fraction on the histological slide in routine pathological diagnostics already serves as a decision tool for or against chemotherapy in hormone receptor positive breast cancer. Nevertheless, inconsistency in Ki-67 assessment in moderately differentiated breast cancer is widely observed and the use of Ki-67 biomarker is controversially discussed as a parameter for treatment decisions in such breast cancer patients. There are some studies so far describing the inconsistency in Ki-67 assessment in routine diagnostic in breast cancer [3, 6–11]. Despite recommendations from the International Ki-67 in Breast Cancer Working Group the inter-observer variability of routine Ki-67 assessment in breast cancer remains poor to moderate especially in the G2 breast cancer group (Kappa: 0.2–0.4) [3, 8–12]. The Swiss Working Group of Breast- and Gyneco-pathologists has surveyed inter- and intra-observer consistency of Ki-67-based proliferative fraction in breast carcinomas and showed good to very good agreement in well (G1) and poorly (G3) differentiated breast carcinomas [3]. However, there was a high degree of inter- and intra-observer inconsistency in the read-outs of Ki-67 (LI) in moderately differentiated (G2) carcinomas, which is particularly problematic because it is for these “intermediate” carcinomas where guidance from the carcinoma’s proliferative activity is expected for chemotherapy decisions [3, 13,1].

In our study we addressed the question whether a systematic further analysis of different counting methods, based on the problematic issues identified in the previous study of the Swiss Working Group of Gyneco- and Breast-Pathologists can improve the unsatisfactory inter- and intra-observer reliability in Ki-67 LI in midrange breast cancer [3]. We systematically analyzed Ki-67 LI on 50 midrange breast cancers applying different counting methods using light microscopy and digital analyses. As endpoint of the study, Ki-67 LI assessment expressed in % according to the different methods was aimed. Intra- and inter-observer reliabilities with these values were calculated and the best reproducible way to assess Ki-67 LI to determine was the end goal of the study.

## Materials and Methods

### Study objective

The main goal of the study was to establish the most reproducible method of assessing proliferative activity (Ki-67 labeling index) in moderately differentiated breast carcinomas (G2), reaching the highest reproducibility.

### Study design

**Fig 1:**
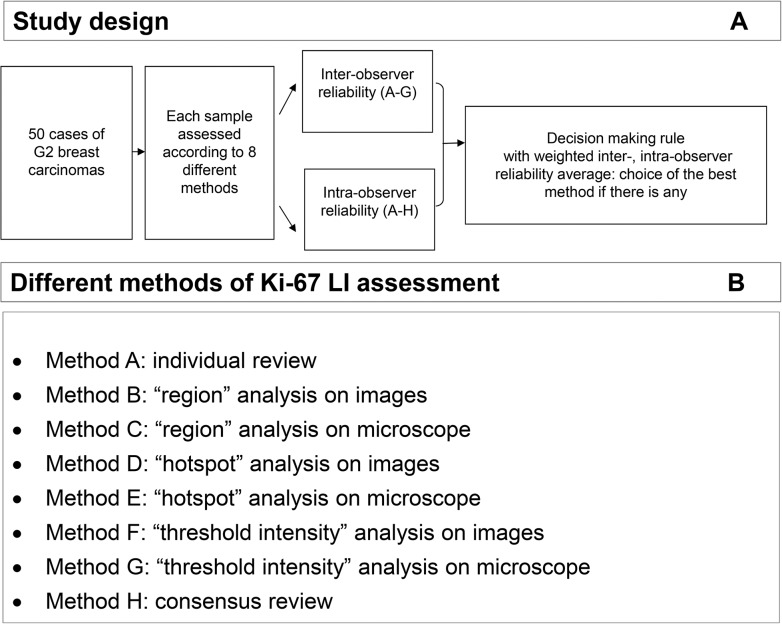
Study design. A) Summary of study design and decision making. B) Summary of methodologies used for the Ki-67 assessment.

Five experienced breast pathologists participated in the study and assessed Ki-67 LI on whole tissue sections containing midrange invasive breast carcinoma following eight assessment methodologies. Exact description of the methods is described below (Assessment methods of Ki-67 LI). The experts were breast pathologists and also members of the Working Group of the Gyneco- and Breast-Pathologist of the Swiss Society of Pathology. Each expert used her/his own light microscope for the methods A, C, E and G. The samples were assessed three times with an interval of at least 2 weeks between the repetitions (**Fig 1**)

### Patients’ cohort

We retrospectively identified 50 breast cancer patients for the study from the Institute of Surgical Pathology, University Hospital Zurich, Switzerland (n = 50). All 50 cancers were moderately differentiated (G2) breast carcinomas by Elston and Ellis [14]. All patients were female, median age was 62.7 years (range: 37 to 83 years). 35 of 50 tumors were histologically invasive ductal carcinomas (no special type, NST), 11 of 50 cases were invasive lobular carcinomas and 4 of 50 cases corresponded to special subtypes (2 mucinous carcinomas, 1 micropapillary carcinoma and 1 metaplastic carcinoma). Except one case (a triple negative metaplastic carcinoma) all carcinomas were hormone receptor positive and Her2 negative. Histological tumor stage was seen as follows: 25 of 50 cases were pT1, 22 of 50 cases were pT2 and 3 cases were pT3. 35 of 50 cases were nodal negative, 15 of 50 cases were nodal positive. 12 of 50 cases were multifocal tumors. None of the patients underwent preoperative chemotherapy. Patients were selected for the study, if postoperative surgical specimen with sufficient invasive carcinoma tissue (minimum 5 mm invasive carcinoma) was available.

This project is a part of a retrospective breast cancer study on archived human tissue material, which was approved by the Ethical Committee of the Canton Zurich (ZH-KEK-2012-553). Additional informed consent was not necessary as the ethical approval covered the ethical issues of the retrospective study and the samples were all anonymized and de-identified prior to the study. The Ethical Committee of the Canton Zurich specifically approved this study including the study protocol.

### Immunohistochemistry for Ki-67 and digital image analysis

The 50 paraffin blocks containing tumor tissue were re-cut and re-stained for Ki-67 according the following laboratory parameters (protocol Institute of Surgical Pathology, University Hospital Zurich, Switzerland, Laboratory for in-situ technology): 2 micrometers slides were cut from paraffin-blocks, which contained formalin fixed tumor tissue. During the whole staining procedure the slides were treated with the fully automated Benchmark staining system (Ventana Medical Systems) using the primary antibody (rabbit monoclonal anti-Ki-67 human, clone 30–09 Ventana Medical Systems, Inc). The immunohistochemichal procedures were carried out at the same time on the empty slides from each paraffin block.

From each tumor sample the participating pathologists received one H&E stain and one Ki-67 immunostain. All Ki-67 immunostains of the principal investigator (Z.V) were scanned in the Institute of Surgical Pathology, University Hospital Zurich. The digital links containing the immunostains were provided to the participating pathologists, which were blinded for the results of the assessment of others. The computer software used for the methods C, E and G is the validated ImmunoRatio web-based software for quantitative image analysis of Ki-67 immunohistochemistry in breast cancer tissue section [15].

### Statistical analysis

Inter- and intra-observer reliability, denoted by ρ_inter_ and ρ_intra,_ were estimated using the approach proposed by Eliasziw et al. [16]. Weighted sum of inter- and intra-observer reliability was calculated for each method using the pre-specified weight factor 0.6 (for inter-observer reliability) and 0.4 (for intra-observer reliability). P-values were calculated based on the confidence bounds.

Additionally, Fleiss’ kappas based on different clinically established cut-offs (14% and 20%) were estimated along with their lower 95% confidence bounds and p-values, which were computed using bias-corrected bootstrap methods.

Multiple testing corrections were not applied on these p-values, thus the results are considered exploratory.

### Determination of sample size

The purpose of this study was to select the best method in terms of concurrent assessment of inter- and intra-observer reliability where 5 raters took repeated measurements in a series of tumor samples. The method proposed by Eliasziw et al. can be applied in this situation, so that “both sources of error (inter-rater and intra-rater) are simultaneously incorporated into the resulting statistical analysis” and “each individual measurement contributes to the estimation of both inter-rater and intra-rater reliability coefficients” [16]. According to the recommendation by Elasziw, for our study 50 study samples, 5 observers and 3 repetitions were more than sufficient to test a reliability hypothesis (H0: ρ_inter_ ≤0.6 versus H1: ρ_inter_ >0.6; H0: ρ_intra_ ≤0.6 versus H1: ρ_intra_ >0.6) at the 5% significance level with 80% power [16], under the assumption that the effect size was 0.15. (**Fig 1A**)

### Assessment methods of Ki-67 labelling index (Fig 1B)

#### Method A: individual review

The percentage of cells showing definite nuclear immunoreactivity among 2,000 invasive neoplastic cells in randomly selected, high-power (magnification, ×400) fields at the periphery of the tumor was calculated on light microscope using the originally recommended criteria [4, 5, 17]. Definite nuclear positivity was defined as any stain independently from the stain intensity as defined by Dowsett et al. and Viale et al. earlier [4, 5, 17]. No hot spots were selected for this analysis.

#### Method B: “region” assessment on image analysis

Comparative analysis of five random fields within the tumor (both periphery and center) (n = appr. 500 cells) using the digital links with Ki-67 stains on the computer screen and the online tool (ImmunoRatio).

#### Method C: “region” analysis on light microscope

Comparative analysis of five random fields within the tumor (both periphery and center) (n = appr. 500 cells) using the own light microscope on 20x magnification.

#### Method D: “hotspot” assessment on image analysis

Analysis of five random fields with the highest index (hotspots) within the tumor (n = appr. 500 cells) using the digital links with Ki-67 stains on the computer screen and the online tool (ImmunoRatio).

#### Method E: “hotspot” analysis on light microscope

Analysis of five random fields with the highest index (hotspots) within the tumor (n = appr. 500 cells) using the own light microscope on 20x magnification.

#### Method F: “threshold intensity” assessment on image analysis

Comparative analysis of five peripheral fields assessing only darkly stained nuclei (n = appr. 500 cells) using the digital links with Ki-67 stains on the computer screen and the online tool (ImmunoRatio).

#### Method G: “threshold intensity” analysis on light microscope

Comparative analysis of five peripheral fields assessing only darkly stained nuclei (n = appr. 500 cells) using the own light microscope on 20x magnification.

#### Method H: consensus review

All slides together are assessed on the multi-head microscope using the three following methods: “no hotspot” (defined above as individual review), “region” analysis on microscope and “hotspot” analysis on microscope. From these 3 methods, one single value was attributed as the Ki-67 index by making a majority approval.

#### The way of Ki-67 assessment

Methods A/C/E/G/H: eyeballing (estimation).

Methods B/D/F: positive and negative Ki-67 areas by image analysis (links to Ki‐67 stains were provided via email, web‐software: ImmunoRatio). The online tool was used by all raters at the standard pre-calibrated basic modus, following the instructions of the software. The area of the histological section was determined by the individual raters based on the definition above. The counting of the areas was done automatically by the software.

**Fig 2:**
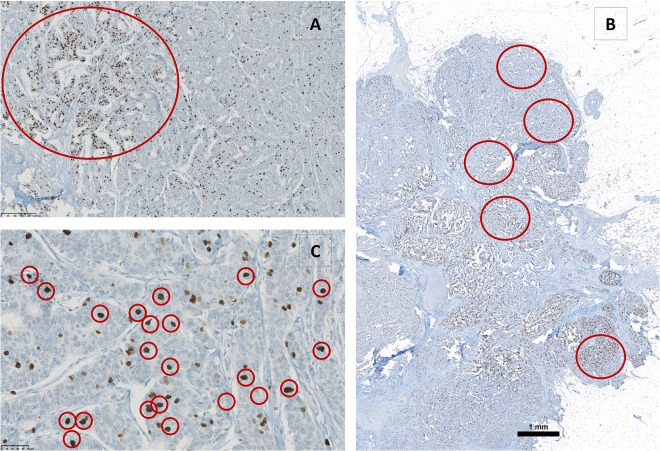
Illustration of different Ki-67 assessment methods. A) Analysis of hotspots. B) Analysis of different areas. C) Analysis of only darkly stained nuclei.

At 40x magnification on the microscope, in solid tumors, there are approx. 300‐400 cells detectable for the measurements. (**Fig 2**)

#### Dropouts

Due to missing assessments from one expert, only assessments from 4 experts were considered in the statistical analysis.

Since method H was very time-consuming and required that all experts were present during the assessment, it was not possible to conduct this assessment and thus method H was not considered for the analysis.

#### Interpretation of ICC and Fleiss’ kappa

The calculation of inter-observer reliability according to Eliasziw et al [16], is a generalized version of the ICC. We used the scale of the ICC to interpret the results. For each method, we estimated the inter- and intra-observer reliability by considering the expert to be random. Two reasonable scales for the interpretation of ICC and Fleiss’ kappa are shown in Table 1.

**Table 1:** Interpretation of ICC from Rosner (A) and Fleiss‘ kappa from Landis and Koch (B).

**A**	
**ICC**	**Interpretation of ICC from Rosner**
**< 0.4**	**Poor agreement**
**0.4–0.75**	**Fair good agreement**
**0.76–1.00**	**Excellent agreement**
**B**	
**Kappa**	**Interpretation of Fleiss‘ kappa from Landis and Koch**
**< 0**	**Poor agreement**
**0.01–0.20**	**Slight agreement**
**0.21–0.40**	**Fair agreement**
**0.41–0.60**	**Moderate agreement**
**0.61–0.80**	**Substantial agreement**
**0.81–1.00**	**Almost perfect agreement**

## Results

The statistical analysis was completed with the data of four experts using seven methods (A—G) with following results:

### Results of inter- and intra-observer reliability (Illustrated in Figs 3 and 4, details shown in Table 2A)

**Fig 3:**
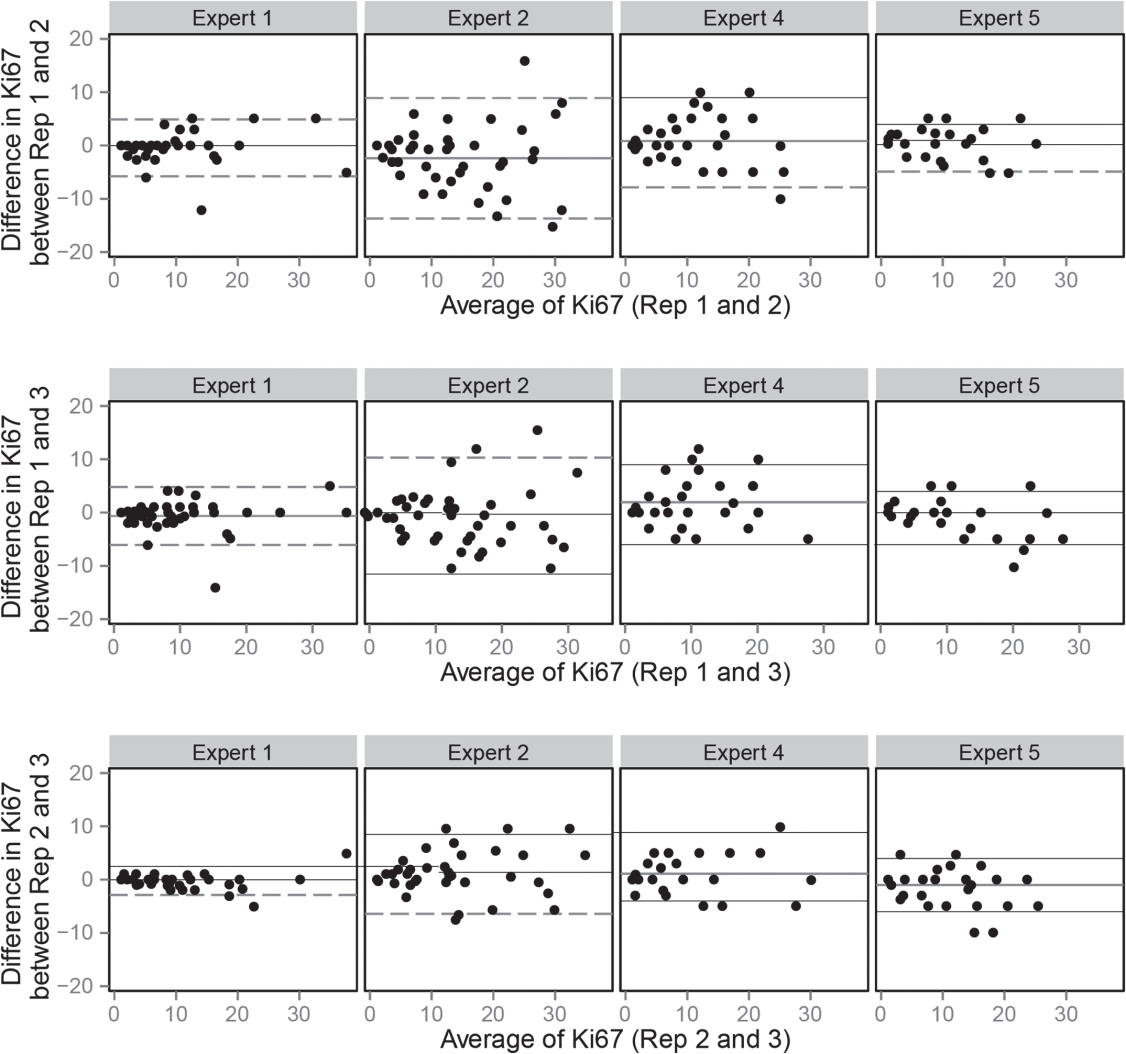
Bland Altmann plots for method A.

**Fig 4:**
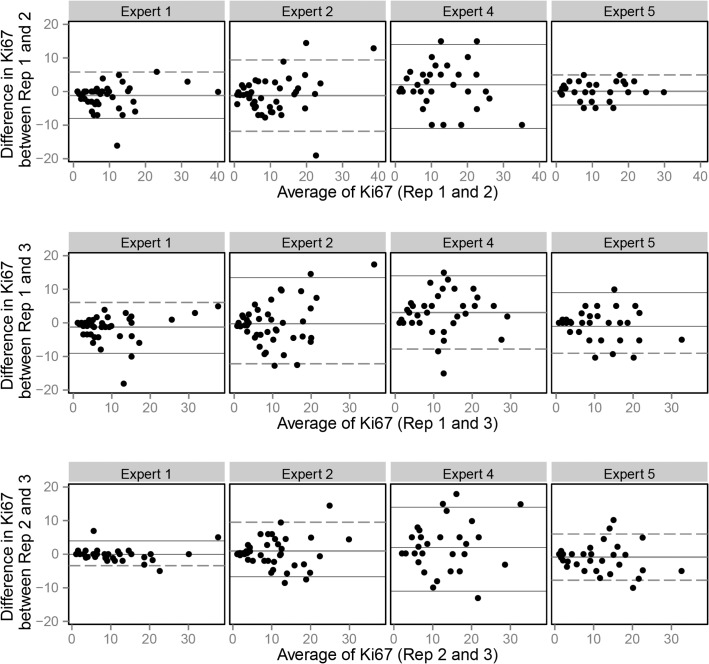
Bland Altmann plots for method C.

**Table 2:** Summary of inter- and intraobserver reliabilities (A) and Fleiss‘ kappas based on different cut-offs (B).

**A**				
**Method**	**Inter-Observer Reliability**	**Intra-Observer Reliability**	**Weighted Sum of inter and intra-observer reliability**	**Ranking ‘best method’**
**A**	**0.71**	**0.88**	**0.778**	1*
**B**	**0.63**	**0.72**	**0.666**	5*
**C**	**0.74**	**0.82**	**0.772**	2*
**D**	**0.57**	**0.69**	**0.618**	6*
**E**	**0.55**	**0.89**	**0.686**	3*
**F**	**0.40**	**0.76**	**0.544**	**7**
**G**	**0.60**	**0.81**	**0.684**	4*
**B**				
**Method**	**Fleiss’ kappa < = 14vs>14**		**Fleiss’ kappa < = 20vs>20**	
**A**	**0.58**		**0.36**	
**B**	**0.51**		**0.37**	
**C**	**0.52**		**0.38**	
**D**	**0.44**		**0.48**	
**E**	**0.40**		**0.48**	
**F**	**0.29**		**0.19**	
**G**	**0.44**		**0.14**	

*: the method reached the requirements of the validation in the next step. The methods whose weighted sum of estimated inter- and intra-observer reliability was larger than 0.6 were in principle qualified to be validated in a further phase of the study.

P-values were calculated based on the null hypothesis that the agreement (Inter- and intra-observer reliability/Fleiss’ kappa) ≤ 0.6.

#### Inter-observer reliability

The inter-observer reliability was fair to good in all methods. The two highest inter-observer reliabilities were measured by methods which were carried out on the light microscope. Method A (individual review on microscope), reaching 0.71 and method C (region analysis on microscope by assessing regions only) reaching 0.74 were the best methods. The highest inter-observer reliability on the image analysis was received by method B (region assessment by image analysis) reaching 0.63.

#### The intra-observer reliability

The intra-observer reliability was excellent in Methods A/C/E/F/G, reaching 0.76–0.89 and it was fair to good in Methods B/D reaching 0.69 resp.0.72.

#### Weighted sum of inter- and intra-observer reliability

Six out of seven methods (A/B/C/D/E/G) reached a minimum of 0.6 as the weighted sum of the inter- and intra-observer variability. The weighted sum of inter- and intra-observer reliability was excellent in methods A and B and fair to good in the other methods.

#### Summary of inter-and intra-observer reliability

The best methods as the result of the weighted sum are methods A (individual review on microscope) and C (region analysis on microscope). None of the methods by image analysis was superior to the methods which were carried out on the microscope.

### Fleiss’ kappa in terms of reproducibility of the clinically used cut-offs of 14% and 20%, respectively (in Table 2B)

#### Fleiss’ kappa in terms of reproducibility of 14% cut-off

The reproducibility of the 14% cut off was moderate in five methods (A/B/C/D/G) and fair in two methods (E/F). The highest value of 0.58 was reached by method A (individual review on microscope).

#### Fleiss’ kappa in terms of reproducibility of 20% cut-off

The reproducibility of the 20% cut off was moderate in two methods (D/E), fair in three methods (A/B/C) and slight in two methods (F/G). The highest value of 0.48 was reached by methods D/E (hotspot analysis on microscope and on image analysis).

#### Summary of Fleiss’ kappa values in 14% and 20% cut-off

In general, slight to moderate agreement was observed, with higher values for the 14% cut-off. The highest Fleiss’ kappa (0.58) was observed for method A (individual review on microscope) with a 14% cut-off.

## Discussion

In our study we addressed the question whether we can identify a methodology for breast cancer proliferative activity using Ki-67 immunohistochemistry with an improved intra- and inter-observer variability. We have set up a design on whole tissue sections and applied seven assessment methodologies using light microscope and digital image analysis. We could show in our study that inter-observer reliability was improved and reached the highest (yet still moderate) value when the analyses were carried out on the light microscope and the methodology included the analysis of regions and of the original recommendation [4, 5]. In terms of reproducibility of clinically relevant and previously established cut-offs for Ki-67 LI the highest agreement of cut-off 14% (yet moderate) was achieved when using light microscope and applying the original recommendation for Ki-67 LI assessment [2, 4, 18]. The clinically recently introduced cut-off of 20% Ki-67, could not superiorly be reproduced in this study, even though analyzing hotspots (both on light microscope and image analysis) reached the highest (yet moderate) reproducibility, which was inferior to the Fleiss’ kappa values achieved at the 14% cut-off. No methodology by image analysis was superior to the methods applied on the light microscope in our study.

The current study and the results represent slight improvement compared to previously published reproducibility studies [3, 8, 9, 11]. These studies, including the first reproducibility study of the Swiss Working Group of Gyneco- and Breast Pathologists, reported poor to fair agreement on Ki-67 LI assessment in midrange breast cancer, with Kappa values ranging from 0.04–0.36 [3, 8, 9, 11]. Based on the first reported data, no single factor of Ki-67 assessment could be attributed to poor reproducibility, however, it was generally the view, that the failure in reproducibility of Ki-67 Li is due to a combination of factors as the area choice for the assessment, intratumoral heterogeneity, differences in preanalytical or laboratory procedures or to the definition what constitutes a positive cell [3]. Other studies pointed out the potential influence of different antibodies on Ki-67 LI. These data however are conflicting in terms of significant influence, varying from relevant to no significant impact on percentage of stained cells [6, 11]. In 2011, the International Ki-67 in Breast Cancer Working Group reported a first recommendation on Ki-67 Li assessment, which addressed also the handling of heterogeneous tumor areas and the presence of as condensed areas, so called hot-spots within the tumor [17]. Despite these guidelines, which incorporated the originally described counting methodologies in Ki-67 LI assessment, there has been no major break-through in terms of a reliable and reproducible method in Ki67 assessment in midrange hormone positive breast cancer until now [4, 17]. The second study of the International Ki-67 in Breast Cancer Working Group conducted a tissue micro array based reproducibility study and showed excellent inter-laboratory and intra-laboratory reliability [10], which very well corroborates our current data on whole tissue sections. Five of the seven methodologies we tested in this study resulted in a good intra-observer reliability. Probably this was due to the fact that pathologists most likely analyze morphological tissue sections and immunohistochemical reactions in a similar yet individual way. As to inter-observer reliability, the study by Polly et al showed that local or central assessment of Ki-67 Li was only moderate (ICC varying from 0.59 to 0.71) even though the study used tissue micro arrays, where knowingly intratumoral heterogeneity poses less importance theoretically [10]. The results on inter-observer reliability in our own current study yielded very similar results, with ICC being 0.73 at highest. It is suffice to say though, that the methods in our study, with ICC >0.60 were achieved on whole tissue sections using light microscopy and considering analyses of different regions. This is most likely explainable in that way that an average value of Ki-67 LI on low-power magnification most likely reflect a doable way to evaluate the whole section and draw an average of the stained cells from the tumor periphery respectively from the invasion front. On the other hand, we need to state that pre-analytical variations were excluded in our study, as all stains were performed in a central laboratory in Zurich, all slides from each block being incubated during the same procedure. Nevertheless, the issue of pre-analytical variables and interpretational differences needs to be kept in mind, when comparing results between different institutions and/or individual raters, as pointed out previously in the literature [3, 19]. None of the methods using digital image analysis were superior to the methods on light microscope in our study.

The reproducibility of clinically relevant cut-offs has been also the subject of some recent studies [19]. In a study of the European Working Group of Breast Screening Pathology, the provided Ki-67 LI-s of the participating institutions were clustered around numbers ending with 0 or 5, questioning the realistic reproducibility of cut-offs different from these numbers [19]. The original recommendation of Ki-67 LI assessment in 2008 set a cut-off of 14% for patients, who benefited from a chemotherapy [4]. Even though the reproducibility of the 14% cut-off poses a diagnostic challenge at the moment, the only method in our study, yielding the highest Fleiss’ kappa values, was the 14% cut-off using the recommendation by Viale at al from 2008 [4]. The recently recommended cut-off of 20% was inferior in the reproducibility for the 14% cut-off in our study [2].

Clinical guidelines increasingly incorporate the potential or recommended use of Ki-67 LI in the clinical oncological decision algorithm, although caution is drawn to the still relevant reproducibility issues in midrange breast cancer in routine histopathological diagnostics [1, 2, 13]. Along with the recommendation of current German guidelines, very similar to our own observations from 2012, the degree of Ki-67 LI can be reliably assessed and reproduced in low- and high ranges, however, caution is needed in mid-range breast carcinomas when dealing with an adjuvant oncological situation [1, 3, 13]. The impact of Ki-67 LI in neoadjuvant setting, especially in triple negative breast cancer, nevertheless has level I evidence and is increasingly applied in core biopsies in the neoadjuvant setting [1, 13, 20].

Genomic tests versus Ki-67 LI as the best method to predict clinical response remains an issue to be further explored [1, 13, 20]. Varying degree of agreement between Ki-67 LI and genomic scores were reported in the literature, which at the current moment addresses the need for further analytical and comparative studies in that field [21–24].

In summary, we could show in our study, that moderate improvement in the inter-observer reproducibility of Ki-67 LI in midrange breast cancer was possible when applying standardized pre-analytical procedures and using light microscopy on whole sections under consideration of regional analyses. None of the methods by chosen image analysis were superior to the measurements on the light microscope. This improvement in inter-observer reliability even though moderate, is promising and needs to be further validated in different patient cohorts and incorporating clinical variables.
